# A Randomized Clinical Trial on Acupuncture Versus Best Medical Therapy in Episodic Migraine Prophylaxis: The ACUMIGRAN Study

**DOI:** 10.3389/fneur.2020.570335

**Published:** 2021-01-15

**Authors:** Giulia Giannini, Valentina Favoni, Elena Merli, Marianna Nicodemo, Paola Torelli, Annunzio Matrà, Carlo Maria Giovanardi, Pietro Cortelli, Giulia Pierangeli, Sabina Cevoli

**Affiliations:** ^1^Department of Biomedical and NeuroMotor Sciences, University of Bologna, Bologna, Italy; ^2^IRCCS Istituto delle Scienze Neurologiche di Bologna, Bologna, Italy; ^3^Department of Medicine and Surgery, Headache Center, University of Parma, Parma, Italy; ^4^Associazione Medici Agopuntori Bolognesi (AMAB) - Scuola Italo-Cinese di Agopuntura Italy, Bologna, Italy

**Keywords:** migraine, acupuncture, prophylaxis treatment, non-conventional treatment, randomized control trial

## Abstract

**Introduction:** A large corpus of evidence has reported encouraging results for acupuncture as a prophylaxis therapy for migraine. However, trials that investigated the efficacy of acupuncture in comparison with pharmacological treatment in episodic migraine showed conflicting results. The study aimed to evaluate if acupuncture is as effective as evidence-based pharmacological drugs in episodic migraine prophylaxis.

**Methods:** This is a randomized controlled clinical study. Patients suffering from migraine without preventive treatment in the past 3 months were recruited. After the run-in period, episodic migraineurs were assigned randomly to two groups: the acupuncture group (A) was treated with 12 sessions of acupuncture, and the pharmacological group (B) was treated with the most appropriate medication for each patient. Headache frequency was compared at baseline and at the end of treatment. Both groups were evaluated 3 and 6 months after treatment.

**Results:** A total of 148 patients (24 males and 124 females) were enrolled in the study. Out of these, 69 were randomized to A and 66 to B. At baseline, no significant differences were found between the two groups. Of the patients, 15.5% (21/135) interrupted the treatment, especially those randomized to B. After 4 months, migraine frequency decreased from 8.58 ± 3.21 to 6.43 ± 3.45 in A and from 8.29 ± 2.72 to 6.27 ± 4.01 in B. Headache frequency decreased significantly after treatment without differences between the two groups (time-effect: *p* < 0.001; group effect: *p* = 0.332; interaction time-group effects: *p* = 0.556). Approximately 34% of patients showed a reduction of headache days by at least 50% after the treatment. The improvements observed at the end of treatment persisted in 57.3% (59/103) after 3 months and 38.8% (40/103) after 6 months, especially in patients randomized to A.

**Conclusions:** Our trial is the first one comparing acupuncture with the more appropriate pharmacological treatment for migraine prophylaxis. Data suggested that acupuncture could be adopted as migraine prophylaxis and seem to be slightly superior to pharmacological treatment in compliance and rate of adverse events.

## Introduction

Migraine is a common disabling primary headache disorder (1), affecting ~15% of adults in Western countries. Its prevalence increases at the age of 35–39 years and in the female sex. It affects adults in an active phase of their life, leading to a significant disability and loss of quality of life, with relevant social and economic costs (2, 3).

The treatment of migraine includes acute therapies that aim to reduce the intensity of pain of each migrainous attack and preventive therapies that should decrease the frequency of headache appearance (4–6).

Despite the great progress in pharmacologic treatment, patients often remain unsatisfied because of the low pain control or the associated unacceptable adverse effects (7).

In the past decades, acupuncture has been pointed out as a valuable non-pharmacological tool in patients with migraine, and its use in clinical practice has been increasing in Western countries. A large corpus of evidence has reported encouraging results for acupuncture as a prophylaxis therapy of migraine (8–10). However, trials that investigated the efficacy of acupuncture in comparison with pharmacological treatment in episodic migraine showed conflicting results, mainly due to differences in population characteristics, study design, and outcome measures (10–16). Moreover, the majority of these studies compared acupuncture with monotherapy as a prophylactic treatment, and findings comparing acupuncture with the best medical treatment are lacking.

## Methods

### Aim, Design, and Setting of the Study

The study aimed to evaluate if acupuncture is as effective as evidence-based pharmacological drugs in migraine prophylaxis. This is a randomized, controlled, open-label, multicenter study. Two Italian Tertiary Headache Centres participated in the study (the Headache Centre of Istituto di Ricovero e Cura a Carattere Scientifico Institute of Neurological Sciences of Bologna and the Headache Centre of the University of Parma).

### Participants

Patients referred to the Headache Centres from 2012 to 2016 were consecutively recruited.

Inclusion criteria for the eligibility in the study were the following: (1) age ≥ 18 years old; (2) ability to give verbal and written informed consent; (3) episodic migraine with and without aura as defined by the International Headache Society (1); (4) absence of preventive treatment in the preceding 3 months.

Exclusion criteria included: (1) severe psychiatric disease; (2) alcohol or drugs addiction; (3) serious ongoing physical illness; (4) inability to sign the informed consent; (5) pregnancy and breastfeeding.

Patients with contraindication to using acupuncture or prophylactic therapies for comorbidities were also excluded.

### Protocol and Visit Assessments

**Figure 1:**
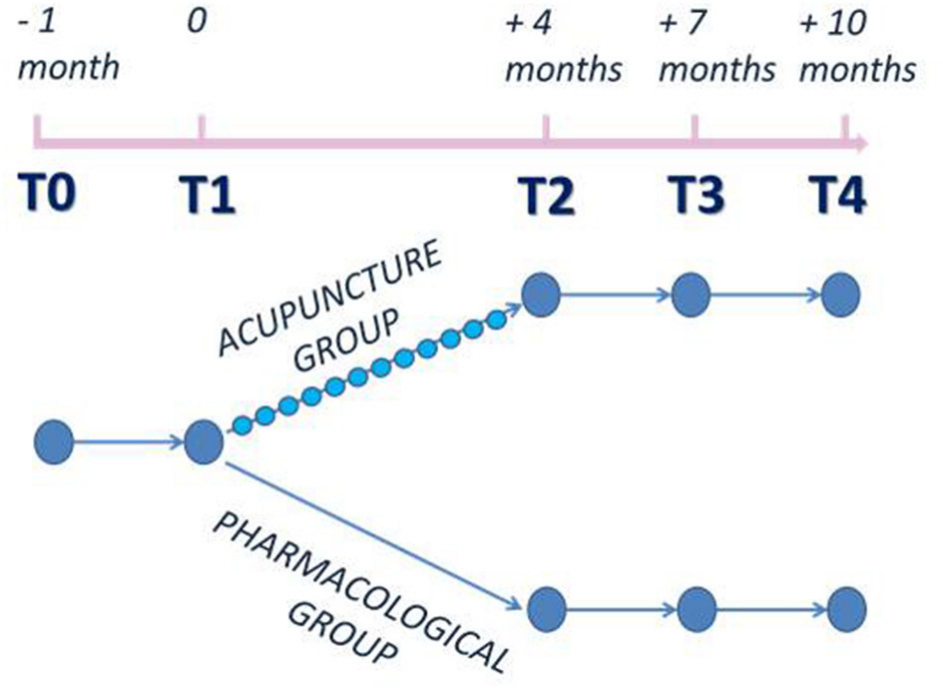
Study design.

Figure 1 illustrates the study design. Visits occurred at baseline (preliminary visit T0), 1 month after baseline (T1), 4 months after treatment program (T2), then at 3 months (T3), and 6 months (T4) after the end of treatment (Figure 1).

Patients with episodic migraine with and without aura without preventive treatment in the preceding 3 months were evaluated in an outpatient visit at baseline (T0). After a 1-month run-in period, eligible patients were randomized (T1) to the acupuncture group (A) or pharmacological group (B) 1:1.

Patients in group A received 12 sessions of acupuncture. The first section included a standardized investigation from an acupuncturist, including a traditional Chinese medicine diagnosis for syndromes. Acupuncture was carried out twice in the first week and weekly for the next 10 sessions and consisted of semi-standardized treatments including some basic obligatory points (LR 3 *taichong*, GB 34 *yanglingchuan*, SP 6 *sanyinjiao*, LI 4 *hegu*, TE 5 *weiguan*, GV 20 *baihui*) and additional individualized points chosen by the physicians on the basis of diagnosis and pain localization (ST 8 *touwei*, BL 2 *zanzhu*; GB 4 *hanyan*, GB 8 *shuaigu*; GB 20 *fengchi*, BL 12 *fengmen*). Acupuncturists were highly qualified medical doctor members of the Associazione Medici Agopuntori Bolognesi—Scuola Italo Cinese di Agopuntura receiving the same training on Acupuncture and Chinese Medicine.

Patients in group B were treated with the most appropriate prophylactic medication for 4 months. Prophylactic treatment was chosen based on the efficacy and adverse effects of previous treatments, comorbidity, and patients' preferences, as in the best clinical practice (4–6). One telephone interview was performed by a trained nurse after 2 months of therapy to investigate compliance and adverse events. All patients were allowed to treat acute headaches as needed. After baseline, concomitant treatment for comorbidities should not be changed.

Subjects were assigned sequentially to group A or B when admitted to the outpatients visit T1 receiving a computer-generated random medication code number. The random allocation sequence was not generated by researchers who assigned participants to interventions.

At T2, prophylactic treatments (both A and B) were stopped. Both groups were evaluated 3 and 6 months after treatment (T3–T4).

A clinical diary in which patients recorded all headache attacks and days, and rescue medication intake for migraine during the study period, was given at T0 and checked at every follow-up visit. At the time of enrolment, patients filled in a questionnaire on preference for acupuncture or pharmacological treatment. Depressive and anxious symptoms (17, 18), degree of disability [Migraine Disability Assessment Score (MIDAS)] (19), and quality of life [36-item Short-Form Health Survey (SF-36)] (20) were evaluated during every visit. At the end of treatment, a satisfaction questionnaire was collected. All patients were interviewed and examined by neurologists with expertise in headaches.

### Outcome Measures

The primary outcome measure was the difference in the number of days with migraine between T1 and T2 as reported by the patient in the headache diary.

Predefined secondary outcomes included: proportion of treatment responders (migraineurs with a reduction of headache days by at least 50% documented in a headache diary), number of migraine attacks, number of rescue medication, number of patients which discontinued the trial, migraine frequency (days and attacks), and rescue medication during follow-up.

### Ethics Approval and Consent to Participate

The study was conducted in agreement with principles of good clinical practice, and the study protocol was approved by the local ethics committee of the local health service of Bologna, Italy (n. 09002). The trial registration requirement in a public trials registry was waived by the ethics committee that approved the study protocol. All patients gave their written informed consent to study participation.

### Availability of Data and Materials

The datasets used and analyzed during the current study are available from the corresponding author on reasonable request.

### Statistics

Normality of continuous parameters distribution was checked using the Skewness–Kurtosis test; variables were expressed as mean ± standard deviation or median along with interquartile ranges when appropriate. Continuous variables were compared by using a *t*-test or Wilcoxon rank-sum, as appropriate. Categorical variables were described by their absolute and/or relative frequencies and compared using the Chi-square test.

Repeated measures analysis of variance was performed to investigate significant main effects for all patients across time. A *p*-value lower than 0.05 (two-sided) was considered significant. Statistical analyses were performed using the statistical software STATA®, version 14.0.

## Results

**Figure 2:**
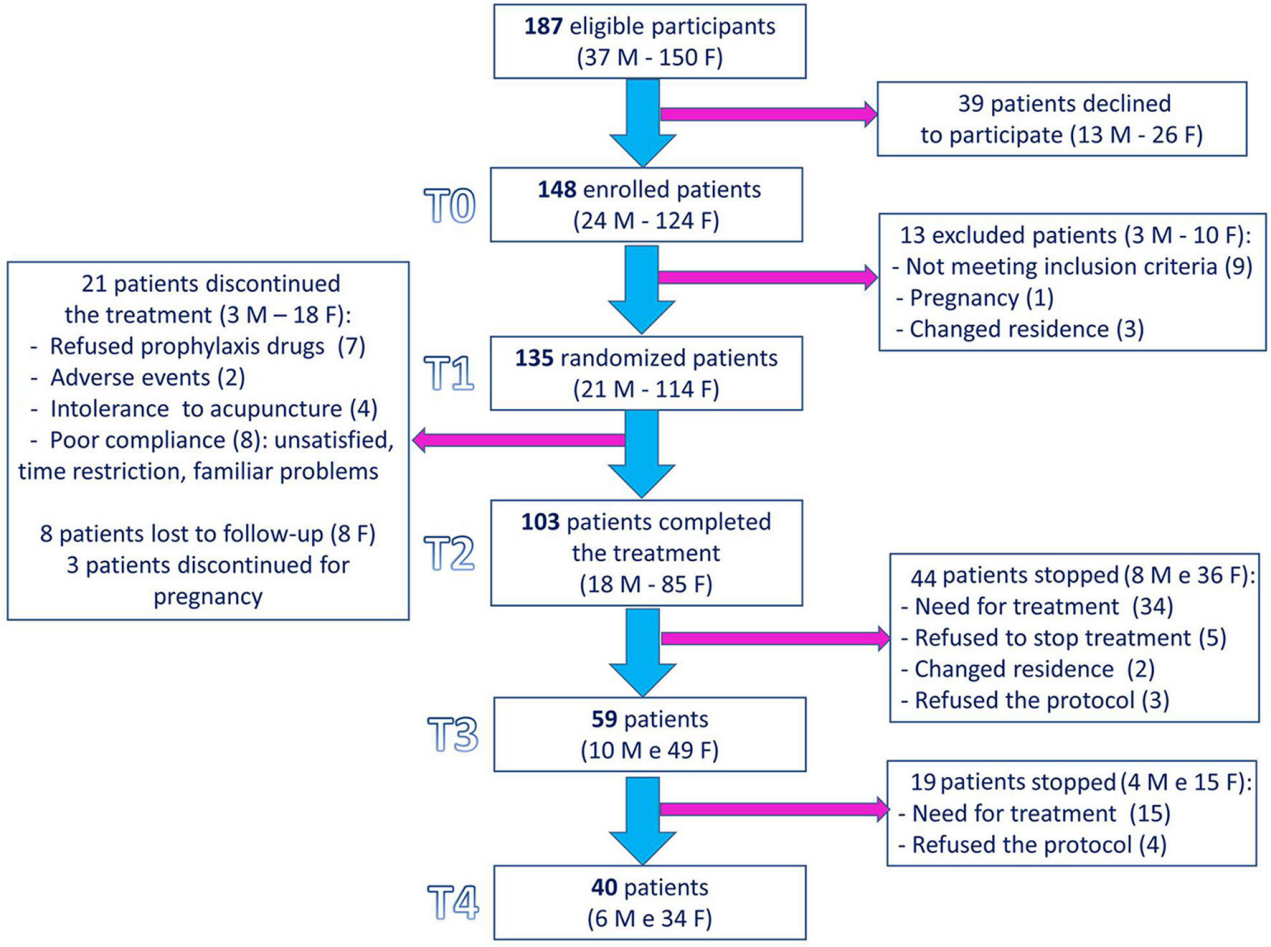
Study flowchart. F: females; M: males.

The study flowchart is shown in Figure 2. A total of 187 consecutive patients (37 males and 150 females) were eligible for the study. Of these, 135 patients (21 males and 114 females) were randomized, and 103 (18 males and 85 females) completed the treatment.

Finally, 59 patients (10 males and 49 females) and 40 patients (six males and 34 females) have undergone the T3 and T4 visits, respectively.

A total of 39 patients (20.9%, 13 males and 26 females) declined to participate in the study: 31 declined because they should not undergo to acupuncture (20 worker patients refused for time reasons, five suffered by fear of needling, three could not participate to acupuncture section for distance reason, one have just been previously treated with acupuncture for migraine, and two should accept only conventional treatment), three declined because they should not intake pharmacological treatment, one declined to participate to a clinical study, and four declined for unknown reasons.

Out of 142 patients enrolled in the study, 13 were not randomized (nine did not meet inclusion criteria, three moved to another town, and one was pregnant).

Out of 135 randomized patients, 32 dropped out: seven withdrew their consent for study participation directly after randomization to B group (refused the prophylaxis drugs), two showed adverse event to drugs, four did not tolerate acupuncture, eight (two patients randomized to A and six randomized to B) showed poor compliance, three (one patient randomized to A and two randomized to B) interrupted treatment for pregnancy, and eight patients (five patients randomized to A and three randomized to B) were lost to the follow-up. The adverse events reported with pharmacological drugs were mild and reversible, but two required the suspension of the treatment: one patient developed depression after the introduction of flunarizine, and another one discovered mild hypertransaminasemia (already presented in the past) and stopped topiramate to investigate further its medical condition.

Out of 135 randomized patients (21 males and 114 females, mean age: 34.2 ± 16.8 years, mean disease duration 24.1 ± 9.9 years), 69 were randomized to A and 66 to B.

Among the patients randomized to B group, 17 (25.8%) received amitriptyline, 7 (10.6%) beta-blockers (4.5% atenolol and 6.1% propranolol), 14 (21.2%) flunarizine, 1 (1.5%) flunarizine + riboflavin (vitamin B2), 9 (13.6%) topiramate, 3 (4.5%) pizotifen, 2 (3.0%) valproic acid, 1 (1.5%) duloxetine + coenzyme Q10, 11 (16.7%) riboflavin (vitamin B2), and 2 (3.0%) a combination of other nutraceutical drugs (magnesium, L-tryptophan, niacin vitamin B2, vitamin D, parthenium, and coenzyme Q10) according to an international guideline (5, 6). The following dosage were used: amitriptyline 25–50 mg/day, atenolol 50–100 mg/day, propranolol 40–80 mg/day, flunarizine 5 mg/day, topiramate 100 mg/day, pizotifen 1–1.5 mg/day, valproic acid 600–1,000 mg/day, duloxetine 60 mg/day, and riboflavin (vitamin B2) 400 mg/day. The demographic and clinical characteristics of the two groups are shown in Table 1. There were no differences in terms of sociodemographic variables, age at migraine onset, disease duration, diagnosis, headache frequency (days and attacks per month), frequency of rescue medications intake (number per month), previous pharmacological and non-pharmacological treatment, scores at Zung scales, MIDAS, and SF-36. Medical conditions did not differ between the two groups. There were no differences in patients' preference questionnaire for acupuncture or pharmacological treatment between the two groups at the time of enrollment.

**Table 1:** Demographic and baseline clinical characteristics of the study sample.

		**Total**	**Treatment Groups**		
			**A: Acupuncture**	**B: Pharmacological**	***p*-value**
**Sample**	*N* (%)	135	69 (51.1)	66 (48.9)	
**Age** (years)	Mean ± SD	34.2 ± 16.8	33.6 ± 17.4	34.7 ± 16.5	0.698
**Sex**					0.899
Males	*N* (%)	21 (15.6)	11 (15.9)	10 (15.2)	
Females	*N* (%)	114 (84.4)	58 (84.1)	56 (84.8)	
**Marital Status**					0.499
Single	*N* (%)	35 (25.9)	15 (21.7)	20 (30.3)	
Married	*N* (%)	88 (65.2)	47 (68.1)	41 (62.1)	
Separated/Divorced	*N* (%)	12 (8.9)	7 (10.2)	5 (7.6)	
Widower	*N* (%)	0 (0.0)	0 (0.0)	0 (0.0)	
**Years of Education**	Mean ± SD	13.6 ± 3.4	13.1 ± 3.4	14.0 ± 3.3	0.127
**Employment**					0.479
Employee	*N* (%)	108 (80.0)	57 (82.6)	51 (77.2)	
Unemployed	*N* (%)	6 (4.4)	1 (1.5)	5 (7.6)	
Housewife	*N* (%)	9 (6.7)	5 (7.2)	4 (6.1)	
Student	*N* (%)	9 (6.7)	4 (5.8)	5 (7.6)	
Retired	*N* (%)	3 (2.2)	2 (2.9)	1 (1.5)	
**Smoke status**					0.710
Nonsmoker	*N* (%)	92 (68.2)	49 (71.1)	43 (65.2)	
Smoker	*N* (%)	18 (13.3)	9 (13.0)	9 (13.6)	
Ex-smoker	*N* (%)	25 (18.5)	11 (15.9)	14 (21.2)	
**Alcohol status**					
No alcohol intake	*N* (%)	44 (32.6)	25 (36.2)	19 (28.8)	0.647
Occasionally	*N* (%)	75 (55.6)	36 (52.2)	39 (59.1)	
Frequent	*N* (%)	16 (11.8)	8 (11.6)	8 (12.1)	
**Age at Migraine Onset (years)**	Mean ± SD	16.2 ± 8.6	16.9 ± 8.2	15.4 ± 8.9	0.2867
**Disease duration (years)**	Mean ± SD	24.1 ± 9.9	23.9 ± 10.0	24.3 ± 9.8	0.8409
**Diagnosis**					
Migraine without aura	*N* (%)	114 (84.4)	55 (79.7)	59 (89.3)	0.063
Migraine with and without aura	*N* (%)	12 (8.9)	10 (14.5)	2 (3.0)	
Migraine without aura + Tension type headache	*N* (%)	9 (6.7)	4 (5.8)	5 (7.6)	
**Previous prophylactic treatment**					0.679
Yes	*N* (%)	72 (53.3)	38 (55.1)	34 (51.5)	
No	*N* (%)	63 (46.7)	31 (44.9	32 (48.5)	
**Efficacy of previous pharmacological treatment**					0.483
Yes	*N* (%)	23 (17.0)	11 (15.9)	12 (18.2)	
No	*N* (%)	44 (35.6)	25 (36.2)	19 (28.8)	
**Efficacy of previous non-pharmacological treatment**					0.330
Yes	*N* (%)	11 (8.1)	5 (7.25)	6 (9.1)	
No	*N* (%)	5 (3.7)	1 (1.45)	4 (4.1)	
**Headache frequency (attacks/month)**	Mean ± SD	5.8 ± 2.2	5.7 ± 2.3	5.8 ± 2.1	0.8079
**Headache frequency (days/month)**	Mean ± SD	8.4 ± 2.9	8.6 ± 3.2	8.3 ± 2.7	0.5700
**Frequency of medication intake (number/month)**	Mean ± SD	8.2 ± 4.5	8.2 ± 4.7	8.3 ± 4.3	0.9263
**Migraine disability assessment score**	Med (IQR)	21; 10–44	20; 14–42	22; 8.5–44.5	0.8317
**Zung Self-Rating Depression Scale**	Mean ± SD	37.3 ± 8.2	37.2 ± 8.6	37.5 ± 7.8	0.8332
**Zung Self-Rating Anxiety Scale**	Mean ± SD	37.2 ± 5.4	37.7 ± 5.4	36.6 ± 5.4	0.2872
**SF-36 Scale**					
Social Role	Med (IQR)	62.5 (50–75)	62.5 (50–75)	62.5 (50–75)	0.7927
Physical Functioning	Med (IQR)	90 (80–100)	90 (80–95)	90 (80–100)	0.6468
Bodily Pain	Med (IQR)	41 (32–51)	41 (32–51)	41 (32–52)	0.5455
Emotional Role	Med (IQR)	66.67 (33.33–100)	66.67 (33.33–100)	66.67 (33.33–100)	0.5705
Physical Role Functioning	Med (IQR)	37.5 (0–75)	25 (0–75)	50 (0–75)	0.4704
General health perception	Med (IQR)	62 (45–77)	62 (45–77)	62 (46–74.5)	0.7193
Mental health	Med (IQR)	64 (52–76)	64 (52–76)	64 (56–72)	0.9080
Vitality	Med (IQR)	55 (40–65)	55 (45–65)	55 (40–62.5)	0.7947

*IQR, interquartile range; med, median; N, sample size; SD, standard deviation; SF-36, 36-item Short-Form Health Survey*.

**Figure 3:**
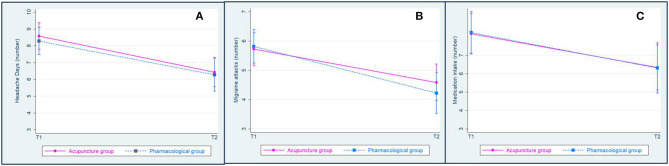
Adjusted predictions of interaction between time (T1 and T2) and groups (acupuncture and pharmacological groups) on parameters with 95% confidence intervals. **(A)** Migraine days; **(B)** migraine attacks; **(C)** medication intake.

The number of headache days decreased significantly after treatment without differences between groups (headache frequency, time-effect: *p* < 0.0001, *F* = 22.61; group effect: *p* = 0.6099, *F* = 0.26; interaction days-group effects: *p* = 0.8768, *F* = 0.02) (Table 2, Figure 3). Responders were 34.78% in the A group and 33.33% in the B one (*p* = 0.477).

**Table 2:** Clinical features at the time of randomization and after treatment of the two groups.

		**Treatment Groups**		
		**A: Acupuncture**	**B: Pharmacological**	***p*-value**
Headache attacks (number/month)				**0.0004**^**a**^ **< 0.0001**^**b**^ 0.6679^c^ 0.4668^d^
T1	Mean ± SD	5.72 ± 2.33	5.82 ± 2.12	
T2	Mean ± SD	4.59 ± 2.74	4.23 ± 2.20	
Headache days (number/month)				**0.0001**^**a**^ **< 0.0001**^**b**^ 0.6099^c^ 0.8768^d^
T1	Mean ± SD	8.58 ± 3.21	8.29 ± 2.72	
T2	Mean ± SD	6.43 ± 3.45	6.27 ± 4.01	
Number of Medication Intake (number/month)				**0.0260**^**a**^ **0.0025**^**b**^ 0.9708^c^ 0.9354^d^
T1	Mean ± SD	8.17 ± 4.71	8.25 ± 4.27	
T2	Mean ± SD	6.34 ± 4.90	6.31 ± 4.54	

a *Repeated measures ANOVA.*

b *From testing parameters for all patients across time (T1, T2).*

c *From testing parameters between groups (acupuncture group and pharmacological group).*

d *From testing the interaction between groups and time of parameters (T1 and T2, acupuncture group and pharmacological group).*

*SD, standard deviation*.

The number of headache attacks decreased significantly after treatment without differences between groups (headache frequency, time-effect: *p* < 0.0001, *F* = 19.03; group effect: *p* = 0.6679, *F* = 0.18; interaction frequency-group effects: *p* = 0.4668, *F* = 0.53) (Table 2, Figure 3). The number of medication intake decreased significantly after treatment without differences between groups (number of acute medication, time-effect: *p* = 0.0025, *F* = 9.38; group effect: *p* = 0.9708, *F* = 0.00; interaction days-group effects: *p* = 0.9354, *F* = 0.01) (Table 2, Figure 3).

According to the intention-to-treat analysis: the number of migraine attacks decreased after treatment without differences between groups; the number of migraine days decreased after treatment without differences between groups; the number of acute medications decreased after treatment without differences between groups.

At the end of treatment, the satisfaction questionnaire and MIDAS score did not differ between the two groups.

Concerning follow-up visit in patients completing the treatment (*n* = 103), 44 patients (18 randomized to A and 26 to B) interrupted the protocol at T2: 34 (18 randomized to A and 26 to B) need to continue the prophylactic treatment due to the frequency of migraine, five (one randomized to A and four to B) preferred to continue their treatment (for other comorbidities as depression, insomnia, etc.), two (one randomized to A and one to B) moved to another town, and three (one randomized to A and two to B) withdrew their consent and refused to continue the protocol. Therefore, 59 patients (39 randomized to A and 20 to B) were evaluated at T3. The frequency of attacks/month was 3.9 ± 2.4 (A: 4.1 ± 2.5, B: 3.5 ± 2.3, *p* = 0.4231), the frequency of days/month was 5.4 ± 3.5 (A: 5.8 ± 3.5, B: 5.0 ± 3.5, *p* = 0.5571), and the number of rescue treatment was 5.7 ± 5.0 (A: 6.3 ± 4.6, B: 4.6 ± 3.4, *p* = 0.2243).

At T3, 19 patients (nine randomized to A and 10 to B) interrupted the protocol: 15 (seven randomized to A and eight to B) need the reintroduction of migraine prophylaxis, and four (two randomized to A and two to B) withdrew their consent and refused to continue the protocol. Therefore, 40 patients (30 randomized to A and 10 to B) ended the protocol. The frequency of attacks/month was 3.7 ± 2.1 (A: 4.1 ± 2.3, B: 2.6 ± 1.4, *p* = 0.0685), the frequency of days/month was 4.8 ± 2.6 (A: 5.2 ± 2.5, B: 3.7 ± 2.8, *p* = 0.1297), and the number of rescue treatment was 4.6 ± 2.9 (A: 5.0 ± 2.9, B: 3.4 ± 2.9, *p* = 0.1769). The two groups did not differ for scores at Zung scales, MIDAS, and SF-36 both at T3 and T4 visits.

On the total sample completing the treatment, 33.0 and 25.4% required prophylaxis therapy after 3 and 6 months, respectively, with a higher proportion in patients randomized to B group (*n* = 19/46, 41.3% after T2; *n* = 8/46, 17.4% after T3) than those randomized to A group (*n* = 15/57, 26.3% after T2; *n* = 7/57, 12.3% after T3).

The improvements observed at the end of treatment persisted after therapy in 57.3% (59/103) after 3 months (T3) and in 38.8% (40/103) after 6 months (T4), especially in patients randomized to acupuncture treatment (68.4% at T3 and 52.6% at T4 in A group; 43.5% at T3 and 21.8% at T4 in B group).

## Discussion

This study suggests that in a population of patients with episodic migraine, acupuncture was as effective as a pharmacological treatment in decreasing migraine frequency. The migraine days in a month decreased significantly after treatment, without differences between the two groups. In the same way, migraine attacks and the number of acute medication significantly decreased after treatment without differences between the two groups. Moreover, in our trial, ~34% of patients showed a reduction of headache days by at least 50% after the treatment.

The analysis of our sample resulted in the further following clinically relevant suggestions: (a) the 15.5% of patients (21/135) interrupted the treatment, especially those randomized to pharmacological drugs; (b) the improvements observed at the end of treatment persisted in 57.3% (59/103) after 3 months and in 38.8% (40/103) after 6 months, especially in patients randomized to acupuncture treatment; (c) the 33.0 and 25.4% required prophylaxis therapy at 3 and 6 months follow-up visits, respectively.

First, our results are in line with previous studies on the effectiveness of acupuncture to standard pharmacologic treatment showing acupuncture to be “at least non-inferior” to conventional treatments in episodic migraine (8–10). However, methodological heterogeneity precludes aggregation of these data and impacts comparison among studies (9, 10). Six previous studies compared acupuncture with pharmacological treatments in episodic migraine (11–16). In the first randomized study, 85 patients with migraine with and without aura were allocated to a 17-week regimen either with acupuncture and placebo tablets or to placebo stimulation and metoprolol 100 mg daily: both groups exhibited a reduction in attack frequency, whereas the metoprolol group showed a lower global rating of attacks (11). In a more recent randomized controlled multicenter trial, 114 migraine patients were randomized to treatment over 12 weeks either with acupuncture (8–15 sessions) or metoprolol (100–200 mg daily). The number of migraine days decreased in both groups, and the proportion of responders (reduction of migraine attacks by ≥50%) was 61% for acupuncture and 49% for metoprolol (16). One randomized controlled trial on 160 women with migraine compared acupuncture (*n* = 80) with flunarizine (*n* = 80) over 6 months found that frequency of attacks and use of symptomatic drugs significantly decreased during treatment in both groups, with a lower migraine frequency after 2 and 4 months in the acupuncture group than in the pharmacological one (12). More recently, a multicenter, double-dummy, single-blinded, randomized controlled trial recruited and assigned 140 patients with migraine without aura to two different groups: the acupuncture group treated with verum acupuncture plus placebo and the control group treated with sham acupuncture plus flunarizine. This study suggested that acupuncture was more effective than flunarizine in decreasing days of migraine attacks, whereas no significant differences were found between the two groups in reducing pain intensity and improvement of quality of life (13).

Another trial was performed in 100 patients with migraine without aura: 50 patients randomized to acupuncture and 50 to valproic acid treatment, during a 6-month follow-up, reported an improvement on MIDAS score during the follow-up in both groups and an improvement on pain intensity and pain relief score in acupuncture group at 6-month visit (14).

Only one prospective, multicenter, double-blind, parallel-group, controlled, clinical trial randomized 960 patients to verum acupuncture (*n* = 313), sham acupuncture (*n* = 339), or standard therapy (*n* = 308): the improvement in the number of migraine days was closely similar in all treatment groups (15).

Second, concerning adverse events and compliance with treatment, 15.5% of patients (21/135) interrupted the treatment (six patients randomized to acupuncture and 15 to pharmacological prophylaxis). Despite no differences in patient's preference for acupuncture or pharmacological treatment between the two groups at the time of enrollment, patients allocated to pharmacological treatment more frequently interrupted the therapy. In particular, in the pharmacological group, seven patients withdrew their consent for study participation directly after randomization, two showed mild adverse events to drugs, and six showed poor compliance, whereas, in the acupuncture group, four did not tolerate acupuncture, and two showed poor compliance. These data suggested that acupuncture showed less adverse effects and higher compliance than pharmacological therapy. Our findings are partially similar to those reported in previous studies. A systematic review published by the Cochrane Library in 2016 found moderate evidence favoring acupuncture over conventional treatment for safety and tolerability, given that acupuncture produced a lower number of pooled adverse effects and had a lower likelihood of dropouts (9). However, previous trials reported a high proportion of participants allocated to drug treatment who withdraw informed consent immediately after randomization [8% (14), 13% (16), and 34% (15)], a high treatment discontinuation [18% (15)] and dropout rates due to adverse effects [9% (12) and 16% (16)]. Compared with other studies, the lower rate of informed consent withdrawn after randomization (10.6%), as well as treatment discontinuation (9.1%) and dropout due to adverse events (3.0%) in our sample, is probably due to the involvement of patients in the choice of the best medical treatment on the basis of their comorbidities (i.e., depression, insomnia, overweight, hypertension, etc.), previous migraine prophylaxis, and their preference to nutraceutical treatment, as in clinical practice (4–6). The higher compliance in the acupuncture group could also be ascribable to a closer follow-up received from patients allocated to acupuncture than those allocated to pharmacological treatment who were evaluated once after 4 months of therapy with a telephone interview after 2 months of treatment. Moreover, in our eligible sample, a large proportion of patients declined to participate at the time of enrolment because they would refuse acupuncture treatment (*n* = 31/39, 79.4%) with respect to those who refused the pharmacological treatment (*n* = 3/39, 7.7%).

Third, 33.0 and 25.4% required prophylaxis therapy during follow-up, especially those randomized to pharmacological treatment. Finally, the improvements observed at the end of treatment persisted after treatment in 57.3% (59/103) after 3 months and 38.8% (40/103) after 6 months, especially in patients randomized to acupuncture treatment. These data suggested that acupuncture could show a prolonged benefit also after session suspension. However, despite a higher dropout rate in the pharmacological group, at a 6-month follow-up visit, patients randomized to conventional treatment showed a lower migraine frequency and acute medication intake without reaching a significant difference. Few studies focused on follow-up efficacy after the end treatment. The study performed by Wang et al. demonstrated that the prophylactic effects of both acupuncture and flunarizine persisted from the end of the treatment through the next 3 months, and verum acupuncture was slightly better than flunarizine in terms of responders proportion (59 vs. 40% after 4 weeks and 56 vs. 37% after 16 weeks, respectively) and mean reduction of migraine days (4.1 vs. 1.9 days after 4 weeks and 4.1 vs. 2.0 days after 16 weeks, respectively) (13). One study compared valproate with acupuncture, showing that both therapies were effective at 3- and 6-month follow-up in relieving migraine regarding the severity of attacks, disability, the intensity of pain, and rizatriptan intake. Valproate provides better control of pain at 3 months, whereas acupuncture is superior at 6 months (14). Similar findings were reported in another study, comparing verum acupuncture with standard drugs after 6 weeks of treatment, in which the responders were significantly higher in the verum acupuncture group (52%) than in the standard drug group (39%) at week 6, without differences between the two groups at week 26 (47% in verum group, 40% in standard group) (15). Therefore, further studies on a larger sample and with a standardized analysis of migraine frequency, adjusted from therapy and other confounding factors, should be performed to better investigate benefit after treatment suspension.

The strength of our study was the semi-structured acupuncture session, the standardized acupuncturist training, and the comparison of acupuncture with the best prophylactic drugs for patients taking into consideration comorbidities (i.e., depression, insomnia, hypertension, etc.) and previous preventive treatment, which probably contribute to improve the compliance and to reduce adverse events.

Several limitations of our study should be discussed. First of all, this was an open study, and the lack of blindness of patients and neurologists could impact the compliance: patients randomized to acupuncture received a closer follow-up, whereas patients randomized to pharmacological treatment were evaluated once after 4 months of therapy. However, we would like to investigate the efficacy of acupuncture in comparison with routine care, choosing the best medical treatment for each patient, as in real-life clinical practice, and for these reasons, we did not take into consideration a study design including sham acupuncture (15, 21–23). The sample size is relatively small, but for feasibility reasons in our neurological ward, we did not recruit further patients. Clinical data of patients were not systematically collected after treatment discontinuation. Finally, the enrollment in tertiary headache centers probably contributed to select patients who preferred traditional treatment, but, on the other side, this allows not to include only subjects with a high expectation for nonconventional treatments.

Our trial is the first one comparing acupuncture with the more appropriate pharmacological treatment for migraine prophylaxis. Data suggested that acupuncture could be adopted as migraine prophylaxis and seem to be slightly superior to pharmacological treatment in compliance and rate of adverse events. However, clinicians should consider that, in our setting, many patients refused acupuncture for time restriction, distance problems, phobia, or preference for standard therapy.

Finally, future researches should be performed to compare acupuncture with the emerging anti-calcitonin gene-related peptide monoclonal antibodies, which have shown comparable efficacy with currently available oral agents for migraine prevention but superior safety and tolerability profiles.

## Data Availability Statement

The raw data supporting the conclusions of this article will be made available by the authors, without undue reservation.

## Ethics Statement

The study was conducted in agreement with principles of good clinical practice and the study protocol was approved by the Local Ethic Committee of the local health service of Bologna, Italy (n. 09002). The requirement for trial registration in a public trials registry was waived by the Ethics Committee that approved the study protocol. All patients gave their written informed consent to study participation.

## Author Contributions

GG: acquisition, analysis, and interpretation of data, and drafting the manuscript. VF, EM, and AM: acquisition and interpretation of data. MN: acquisition of data. PT and CG: acquisition and interpretation of data and critical revision of the manuscript. PC and GP: substantial contributions to conception and design of the study and critical revision of the manuscript. SC: conception and design of the study, acquisition and interpretation of data, supervision of the study, and critical revision of the manuscript. All authors contributed to the article and approved the submitted version.

## Conflict of Interest

The authors declare that the research was conducted in the absence of any commercial or financial relationships that could be construed as a potential conflict of interest. The reviewer PB declared a past co-authorship with several of the authors SC and PC to the handling editor.
